# Digital Interventions to Reduce Distress Among Health Care Providers at the Frontline: Protocol for a Feasibility Trial

**DOI:** 10.2196/32240

**Published:** 2022-02-16

**Authors:** Binh Nguyen, Andrei Torres, Walter Sim, Deborah Kenny, Douglas M Campbell, Lindsay Beavers, Wendy Lou, Bill Kapralos, Elizabeth Peter, Adam Dubrowski, Sridhar Krishnan, Venkat Bhat

**Affiliations:** 1 Department of Electrical, Computer, and Biomedical Engineering Ryerson University Toronto, ON Canada; 2 maxSIMhealth Group Ontario Tech University Oshawa, ON Canada; 3 Interventional Psychiatry Program St Michael’s Hospital Unity Health Toronto Toronto, ON Canada; 4 Department of Nursing University of Colorado Colorado Springs, CO United States; 5 Neonatal Intensive Care Unit St Michael's Hospital Unity Health Toronto Toronto, ON Canada; 6 Allan Waters Family Simulation Program St Michael's Hospital Unity Health Toronto Toronto, ON Canada; 7 Li Ka Shing Knowledge Institute Unity Health Toronto Toronto, ON Canada; 8 Department of Pediatrics Faculty of Medicine University of Toronto Toronto, ON Canada; 9 Department of Physical Therapy Temerty Faculty of Medicine University of Toronto Toronto, ON Canada; 10 Dalla Lana School of Public Health University of Toronto Toronto, ON Canada; 11 Lawrence S Bloomberg Faculty of Nursing University of Toronto Toronto, ON Canada; 12 Department of Psychiatry University of Toronto Toronto, ON Canada

**Keywords:** virtual reality, mobile app, moral distress, simulation, moral injury, COVID-19

## Abstract

**Background:**

Stress, anxiety, distress, and depression are high among health care workers during the COVID-19 pandemic, and they have reported acting in ways that are contrary to their moral values and professional commitments that degrade their integrity. This creates moral distress and injury due to constraints they have encountered, such as limited resources.

**Objective:**

The purpose of this study is to develop and show the feasibility of digital platforms (a virtual reality and a mobile platform) to understand the causes and ultimately reduce the moral distress of health care providers during the COVID-19 pandemic.

**Methods:**

This will be a prospective, single cohort, pre- and posttest study examining the effect of a brief informative video describing moral distress on perceptual, psychological, and physiological indicators of stress and decision-making during a scenario known to potentially elicit moral distress. To accomplish this, we have developed a virtual reality simulation that will be used before and after the digital intervention for monitoring short-term impacts. The simulation involves an intensive care unit setting during the COVID-19 pandemic, and participants will be placed in morally challenging situations. The participants will be engaged in an educational intervention at the individual, team, and organizational levels. During each test, data will be collected for (1) physiological measures of stress and after each test, data will be collected regarding (2) thoughts, feelings and behaviors during a morally challenging situation, and (3) perceptual estimates of psychological stress. In addition, participants will continue to be monitored for moral distress and other psychological stresses for 8 weeks through our Digital intervention/intelligence Group mobile platform. Finally, a comparison will be conducted using machine learning and biostatistical techniques to analyze the short- and long-term impacts of the virtual reality intervention.

**Results:**

The study was funded in November 2020 and received research ethics board approval in March 2021. The study is ongoing.

**Conclusions:**

This project is a proof-of-concept integration to demonstrate viability over 6 months and guide future studies to develop these state-of-the-art technologies to help frontline health care workers work in complex moral contexts. In addition, the project will develop innovations that can be used for future pandemics and in other contexts prone to producing moral distress and injury. This project aims to demonstrate the feasibility of using digital platforms to understand the continuum of moral distress that can lead to moral injury. Demonstration of feasibility will lead to future studies to examine the efficacy of digital platforms to reduce moral distress.

**Trial Registration:**

ClinicalTrials.gov NCT05001542; https://clinicaltrials.gov/ct2/show/NCT05001542

**International Registered Report Identifier (IRRID):**

DERR1-10.2196/32240

## Introduction

### Background

Health care providers, particularly during the COVID-19 crisis, have reported acting in ways that are contrary to their moral values, integrity, and professional commitments because of constraints in their work environments [1,2]. Moral suffering can ensue when a health care provider’s moral foundation is threatened or violated by witnessing or participating in decisions or actions that degrade their integrity [3]. Moral distress might arise when health care providers are unable to translate their moral choices into action because of internal or external constraints [4].

Moral injury, a more extreme form of moral distress, arises in high-stakes situations wherein integrity and conscience have been violated or one’s moral core is eroded [5]. As these events and other workplace stressors accumulate, health care providers’ capacities to provide the level of care they desire can be diminished. There has been a growing attempt to conceptualize moral distress and injury in different health care professions, but the numerous definitions can confuse the issue [6,7]. The concept of moral injury has existed for much longer than its first conceptualization by Shay [7] concerning the military, where it was defined as a betrayal of moral character, usually as a result of the actions of a person in a position of authority. There is a large body of literature within the military context, and moral injury has conceptually evolved to include betrayal of one’s moral values, and furthermore, as a spiritual, psychological, and somatic response to a series of morally injurious events [5]. While some may contend that actions by military members during a war require forethought based on training, others suggest that actions are made on rapid and subconscious decision-making and that moral injury occurs only when individuals question their actions after the fact [8].

However, moral injury is not well defined in health care professions. Cartolovini et al [9] argue that moral injury occurs in health care providers when they encounter traumatic events that involve high-stakes situations beyond their control in which they perpetuate, fail to prevent, or bear witness to actions that transgress deeply held moral beliefs [10]. A related concept, moral distress, was first described within the nursing context by Jameton [11] as “… when one knows the right thing to do, but institutional constraints make it nearly impossible to pursue the right course of action.” Additional studies [12] have provided more context to this definition and have described causes of moral distress as beyond the organization to specific clinical situations and internal caregiver dynamics. Moral distress and injury likely occur along a continuum, with moral injury at the extreme end [13].

The COVID-19 pandemic has necessitated fundamental changes to health care provision. As we continue to learn about the virus and differing courses of disease, health care providers have often willingly accepted inherent risks during the pandemic to care for their patients. However, the need for rapid and changing contingency planning, shortages in personal protective equipment, resource shortages, and the inability to provide the ethical caring called for by professional codes of ethics have placed health care providers in what has been described as a “war zone [14].” The sudden and unprecedented similarities between civilian health care and military scenarios have potentially resulted in moral distress and possibly moral injury. It is nearly impossible to anticipate the course of the virus and make predictions about its physiological outcomes; it is also challenging to predict the course of mental health needs of health care providers, but it is known that there will be needs.

Research has concentrated on treatment for moral injury, either in the military realm or the civilian realm. Gilligan [15] discussed moral injury as “a shattering of trust that compromised our ability to love” and would seem to suggest the health care providers who suffer moral injury during extreme circumstances such as a pandemic may lose the ability for empathy that is required by professional values. Moral injury has been found to not generally respond to typical treatment of posttraumatic stress disorder, even though most treatment focuses on the posttraumatic stress disorder symptomatology [5]. Being able to preserve or restore integrity requires that health care providers have the opportunity for moral repair by strengthening their moral compass and restoring their integrity. Having a moral compass has been identified as a modifiable factor effective in healing from trauma [16]. When one’s moral foundation is clear and accessible, health care providers have a potential opportunity to heal the wounds caused by the COVID-19 crisis and an additional resource that they can leverage when ethical challenges arise.

In this paper, we propose using digital platforms, specifically virtual reality and mobile apps, to develop an understanding of the continuum of moral distress that leads to moral injury in health care workers. Virtual reality technology will be used as a digital platform to simulate an intensive care unit during the pandemic. Our aim is to determine the feasibility of creating a digital environment and implementing digital interventions to better understand the phenomenon of moral distress by developing platforms to examine moral distress and monitor participants over time in order to develop a better understanding of the continuum of moral distress to moral injury.

## Methods

### Ethics

As of March 20, 2021, the research ethics board at St. Michael’s Hospital, Unity Health Toronto has approved the study, and the reference number is UHTDTS25377. The clinical trial is registered with ClinicalTrial.gov with the Registration/identifier number NCT05001542.

### Overview

**Figure 1:**
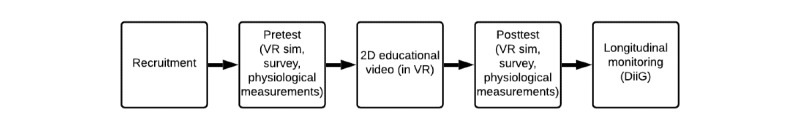
Flow diagram of feasibility trial design. VR sim: virtual reality simulation. DiiG: digital intervention/intelligence group.

We will recruit health care workers to participate in our feasibility trial that will be composed of pretesting, 2D educational video, posttesting, and longitudinal monitoring (Figure 1). During pre- and posttest components, we will pilot unique digital technology platforms (virtual reality environments) to identify circumstances that have the potential to cause moral distress; examine interactions at the individual, team, and organizational levels; perform detailed concept mapping to understand features of moral distress and injury and to aid the design of potential interventions. The educational video will be in 2D; however, the rest of the study components will be 3D virtual reality. The video will be presented on the virtual reality headset after the pretest and before the posttest, and is a brief educational video about moral injury. After the posttest, for 2 months, we will longitudinally monitor participants through the collection of active and passive data through our novel mobile platform.

The expected outcomes of our protocol include (1) using virtual reality technology to design a virtual reality environment to simulate intensive care unit settings during the pandemic and determine the feasibility of understanding moral distress and injury for potential future implementations of digital interventions and (2) using acute physiological monitoring within a virtual reality for simulation of a hospital environment where health care workers have to make challenging moral decisions in complex moral simulated situations, and using a novel mobile platform for longitudinal monitoring by collecting passive and active data to understand the contribution of moral distress to the experience in real-time. The information from these digital platforms will influence each other and aid in the development of models to predict and respond to risks associated with moral distress and injury.

### Experimental Flow

#### Participant Recruitment

Eligible participants include health care providers at 3 affiliated hospitals at Unity Health Toronto, who are 18 years of age or older and who own a mobile phone (Android phones with OS version 6.0 and above, iPhone 6 with OS 11 and above). Since this is a feasibility study conducted among health care providers during a pandemic, we require participants to be currently providing health care at the respective hospitals.

We aim to recruit 15 health care providers (minimum of 10 participants including physicians, nursing, and allied staff) from St. Michael’s, St. Joseph, and Providence hospitals by posting study notices on Unity Health Toronto microsites (eg, Twice a Week newsletters, MD matters, Leadership links), posting flyers at the hospital sites, and by sending email notices. Interested participants will email the study coordinator indicating their interest to participate in the study. The study coordinator will send the participant the consent and demographics forms to sign and complete, and the available time slots to participate in the virtual reality and wearable component of the study. (The demographic survey will include questions regarding age, sex, type of work at Unity Health Toronto, COVID-19 clinical status). Participants will be required to email back their choice for their preferred time slot, the signed consent form, and the completed demographics form.

#### Introduction and Prebrief

The session begins with an introduction of our feasibility trial, goals, and objectives. The session will prepare the participant for the virtual reality simulation in which they take on the role of a health care provider during the COVID-19 pandemic. We will utilize virtual reality simulation to understand the decision-making and emotional response of health care professionals in morally challenging environments, and we will use physiological sensors to monitor the responses. This information will allow us to develop a better understanding of moral distress and create an appropriate intervention.

#### Pretest: Virtual Reality Scenario

After the introduction, participants take on the role of health care providers. The scenario will take place in a hospital setting and will include a physician and nurse, all of whom will be computer-controlled nonplayable characters. There will be an ongoing discussion among them in a scenario that involves decision-making in a morally challenging context.

The virtual reality scenario was created with a particular focus on 2 situations and modified iteratively based on the concepts and research related to moral distress and injury—not having the power to decide which patient to provide life-saving care to when resources are limited and not being able to provide optimum care to all patients.

This pretest virtual reality scenario will initially include all the suggested interventions at the individual, team, and organizational levels and examine the efficacy of these suggested interventions. Additional interventions will be added based on user experience.

#### Intervention (2D Educational Video)

Participants will watch an educational video that effectively summarizes the causes and symptoms of moral distress and injury and the potential interventions at the individual, team, and organizational levels.

#### Posttest: Virtual Reality Scenario

For the posttest, the virtual reality scenario with moral distress and injury knowledge and its respective interventions are repeated.

#### Debrief

After completing the pre- and posttest, participants will engage in a debrief led by the researchers. The debrief includes open-ended questions that will encourage the participants to speak about their experience in the virtual reality setting, followed by an exit survey. If needed, participants will be able to speak with a psychotherapist at the Center for Depression and Suicide Studies at St. Michael’s Hospital.

#### Longitudinal Monitoring

After completing the session, participants will use the app for longitudinal monitoring by collecting passive and active data to understand the distress experienced in real-time.

### Compound Intervention

The compound intervention is composed of the virtual reality simulation-based educational intervention and data collection of mental health and moral injury surveys. The virtual reality simulation-based educational intervention contains a moral injury educational video based on the Moral Injury Guide developed by the Ottawa PTSD Center for Excellence [17].

The latter component of the compound intervention evaluates the effectiveness of the virtual reality simulation-based educational intervention through mental health surveys. These surveys include the Perceived Stress Scale (PSS), Moral Injury Outcome Scale (MIOS), Igroup presence questionnaire (IPQ), and Adapted Moral Injury Symptom Scale: Health care Professionals Version (MISS-HP). PSS is a self-report instrument that evaluates level of stress. MIOS and MISS-HP are tools that evaluate the effect of moral injury, and the IPQ is a questionnaire that evaluates the experience of presence during virtual reality simulations. We will use this information to improve our compounded intervention for use on digital platforms iteratively.

### Trial Design

**Figure 2:**
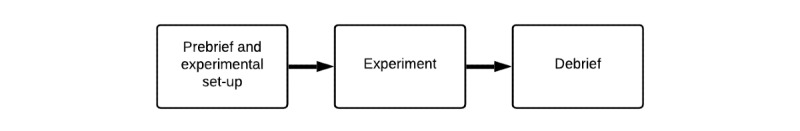
Overview of the virtual reality experimental set-up and data collection.

**Figure 3:**
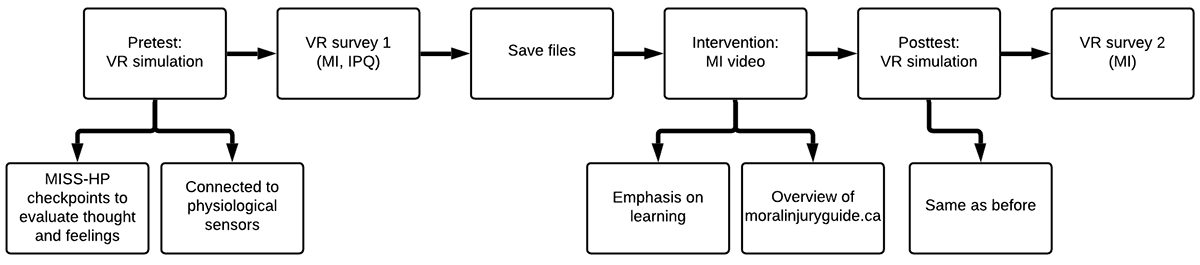
Diagram of experiment components. VR: virtual reality. MI: moral injury. IPQ: igroup presence questionnaire. MISS-HP: moral injury symptom scale: healthcare professionals version.

This is a multilayer feasibility trial (Figure 2). The in-person session (Figure 3), begins with participant arrival at St. Michael’s Hospital Simulation Centre. This is followed by experimental set-up and prebrief. The prebrief is held to inform the participant of the purpose of the session, to collect presurvey data, and obtain written consent. The experimental set-up involves the attachment of the virtual reality equipment and physiological sensors. The debrief is hosted after the experiment and is composed of a semistructured interview, open-ended questions, and an exit survey. The purpose of the debrief is to obtain qualitative feedback on the session to better understand the participant experience.

The virtual reality session will be used for short-term monitoring of participants for acute distress. After the intervention, participants will use a mobile app (developed by the Digital intervention/intelligence Group) for long-term distress monitoring.

### Security and Privacy of the Study Data

During the session hosted at St. Michael’s Hospital Simulation Centre, data (CSV files, text files, audio, and video files from the session) will be securely uploaded to a password-protected drive. Only specific members of the project have access to the data. Responses on paper questionnaires will be input to a spreadsheet and stored on the encrypted drive. During the longitudinal monitoring part of the study, all active and passive data will be stored on the participant’s phone (if storage space is available) until those data are synced back to the back-end server. Data are transferred over a TLS layer; hence, it is encrypted by RSA Asymmetric Encryption Algorithm.

Study data will be stored for 10 years. No personal information will be collected through the mobile component of the study. Demographic data (ie, age, sex, type of job at Unity Health Toronto, diagnosis, and COVID-19 status) will be collected separately from data collected by the app. A study participant ID will link these data for final analysis.

The server and the dashboard will be hosted on the same virtual machine server, and the database will be hosted on a different database server (Multimedia Appendix 1).

### Virtual Reality Development

The first stage of the development involved defining the context of the scenario, with the support of a subject-matter expert, and determining how it connects to the domains *betrayal of the team*, *transgression of moral values*, *emotional response*, and *support and reframing in relation to guilt and loss of trust in colleagues and shame*.

An overview of the scenario was then outlined, describing the location, main characters, key points (eg, the removal of the ventilator), and the actions and reactions of each character. Based on this document, 2 activities were performed in parallel: the definition of the scenario script with the lines and actions of all characters and the development of the 3D assets (characters, objects, and environment) of the virtual reality scenario. Both activities were developed iteratively, with discussions and demonstrations during weekly meetings. The final version of the script includes 7 nonplayable characters (Table 1) and 4 conversations (Table 2), not including the initial tutorial.

**Figure 4:**
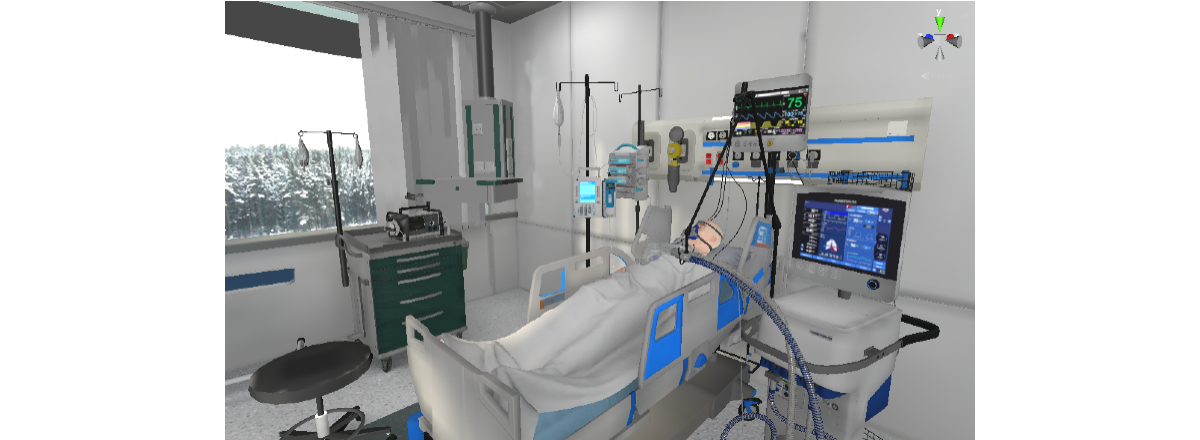
Patient’s room with a patient connected to a ventilator.

**Figure 5:**
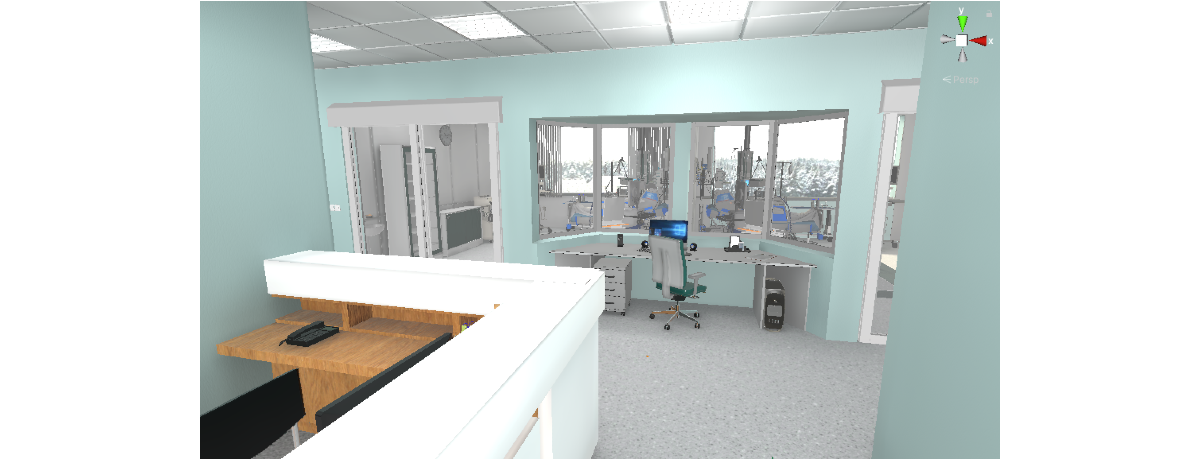
Nursing station, monitor station, and intensive care unit corridor.

For the virtual environment (Figure 4 and Figure 5), royalty-free models and scenarios were purchased and used as a starting point. They were combined and modified to fit the project's needs. Subject-matter experts provided feedback on key elements to make the environment appear closer to the reality of a Canadian hospital environment. The goal was to represent a standard intensive care unit with individual rooms for patients and a nursing station.

**Table 1:** List of nonplayable characters.

Character	Description
Administrator	Intensive care unit team lead
Nurse	Intensive care unit nurse
Mr. Adam	Mr. Adam, a 65-year-old man who has multiple comorbidities with COVID on a ventilator
Mr. Adam’s partner	Distraught, accusing the participant of cruelty, mismanagement, and malpractice
Ms. Betty	A 56-year-old woman has a more favorable prognosis
Medical Doctor 2	Another intensive care unit physician, not aware of the situation
Medical Doctor X	Highly experienced and respected intensivist whose advice and counsel are often sought

**Table 2:** List of conversations.

Scene	Nonplayable characters	Description
Intensive care unit hallway	Administrator, nurse	Conversation between the participant, administrator, and nurse about the lack of ventilators. It is decided to move the ventilator from Mr. Adam to Ms. Betty.
Mr. Adam’s room	Mr. Adam, Mr. Adam’s partner	The participant must inform Mr. Adam’s partner about the decision to remove the ventilator.
Intensive care unit hallway 2	Medical doctor 2	A medical doctor questions the participant about what happened, and they witness Mr. Adam (now without the ventilator) going into cardiac arrest
Nursing station	Medical doctor X	An experienced medical doctor reaches out to the participant to provide counsel and provide support.

**Figure 6:**
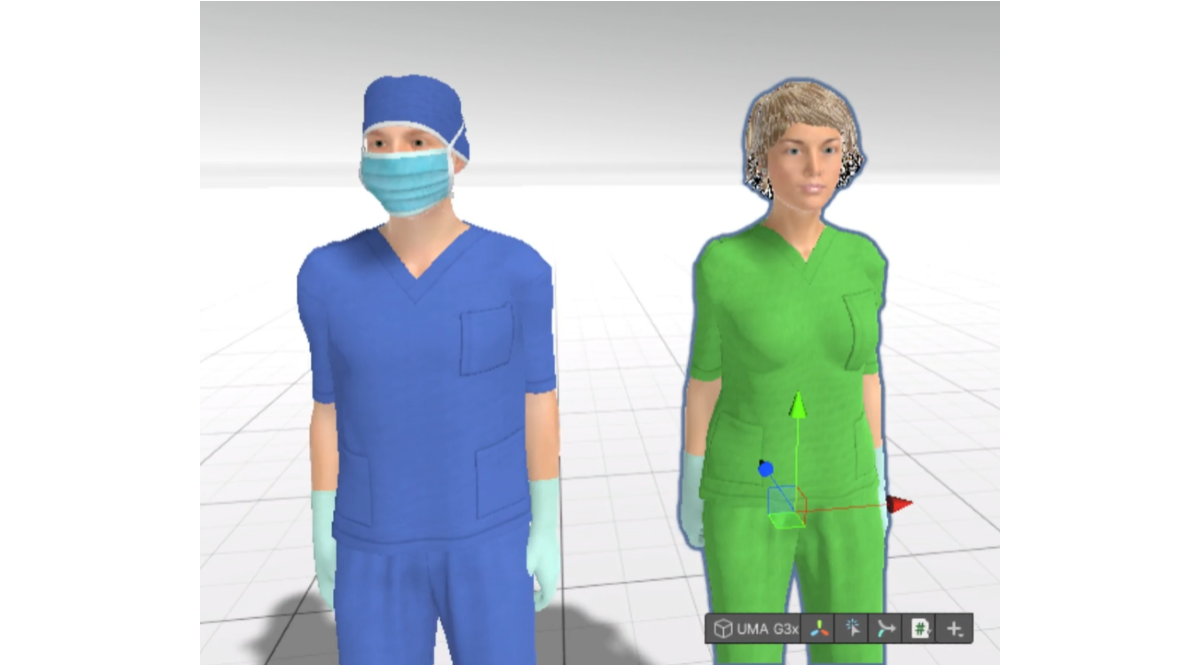
Testing 2 characters with different clothing and body parameters.

To represent the nonplayable characters and the participant, royalty-free models were purchased as starting points. Based on these models, a customizable character system was implemented that allowed us to quickly modify external elements (such as clothing or masks) or the character’s characteristics, such as height and weight (Figure 6).

**Figure 7:**
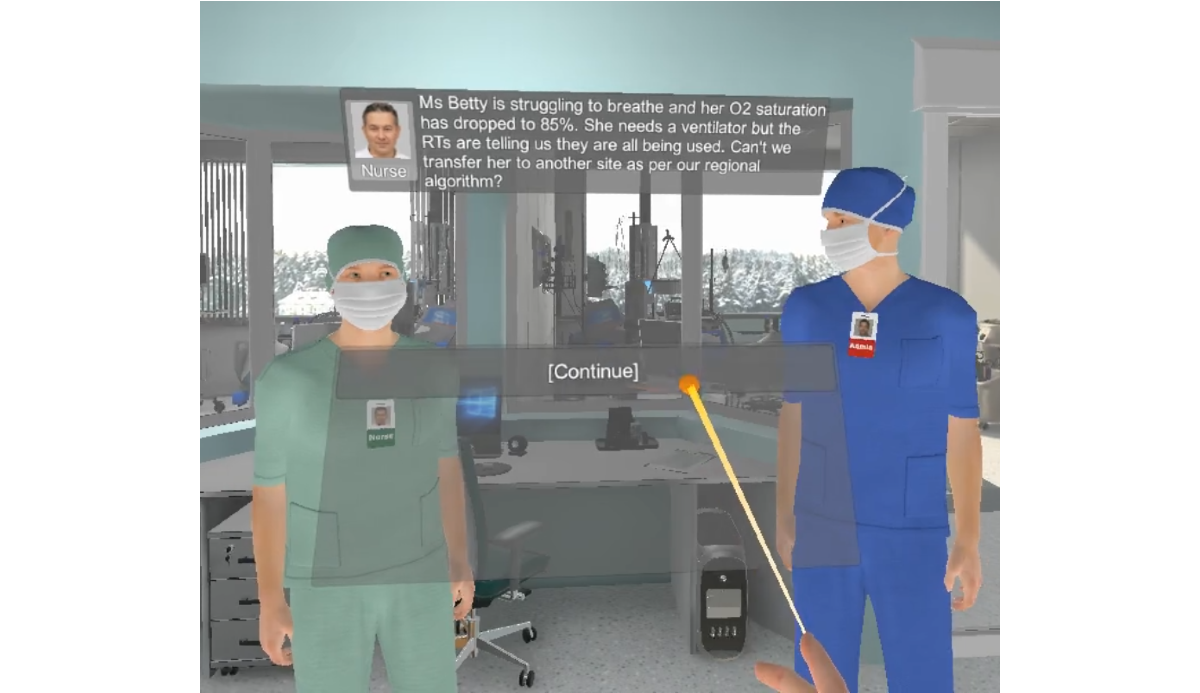
User interface displaying the dialog and interaction panels, and the interaction ray from the right-hand index finger.

For the user interface, 2 semitranslucent panels are displayed as spatial elements in the virtual reality environment (Figure 7). The dialogue panel shows current nonplayable character’s photograph, name, and the text version of the dialogue being spoken. The interaction panel displays available choices (for example, the option *Continue* to advance to the following dialogue or a list of choices).

To interact with the panel, participants need to move one of the controllers, point to the desired choice, and click any buttons under the index finger or thumb. The participant can see a blue ray being casted from their index finger tip within the virtual reality environment, which turns yellow when hovering over the interactive panel (Figure 7). As additional visual feedback, dialogue choices include a highlight effect when the ray hovers over each.

### Software and Hardware

**Figure 8:**
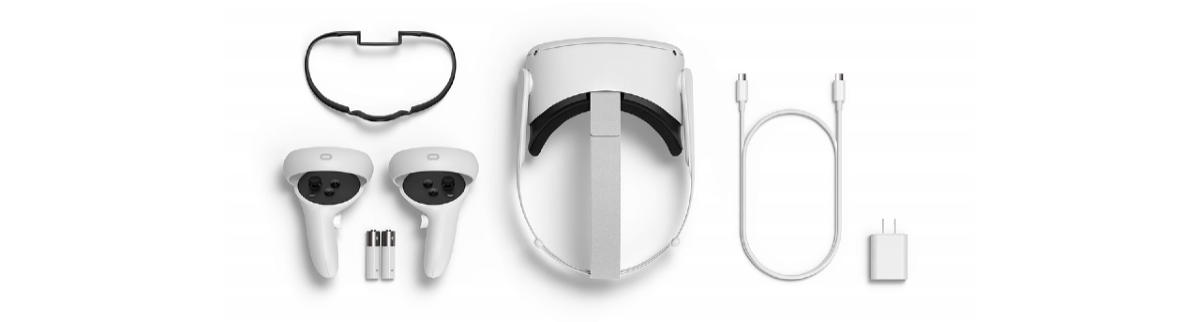
Oculus Quest 2 components.

The virtual reality scenarios were created using a cross-platform game engine (Unity 3D, Unity Technologies) that has been adopted, not only by the game industry, but also by film, architecture, and education industries [18]. The virtual reality scenario is played using a consumer-grade head-mounted display (Oculus Quest 2, MetaQuest) (Figure 8) that can track the user’s head movements with 6 degrees of freedom and track the user’s movements either in stationary mode (sitting in a chair) or in room mode (recommended room size is up to 2 m × 2 m), allowing the user to physically walk around within the defined boundaries. In addition, the system includes 2 handheld motion-tracked controllers that allow the user to interact with the environment and with virtual reality interface components. Our virtual reality scenarios will adopt stationary-mode tracking and use the handheld motion-tracked controllers for more accurate tracking.

Currently, Oculus Quest and Oculus Quest 2 are not Health Insurance Portability and Accountability Act–compliant. However, no data will be stored in the virtual reality device, and the Oculus framework is not used to develop our virtual reality app. The Oculus Quest 2 is connected to a virtual reality-capable PC laptop using a USB-C cable.

### Subjective Data Collection

We plan to collect subjective data such as qualitative data from surveys and open-ended feedback. As mentioned in the previous sections, the MISS-HP, IPQ, PSS, and MIOS will be answered throughout the experiment. At the end of the virtual reality session, an exit survey composed of open-ended and 3 multiple choice questions for general feedback.

Overall, the MIOS and PSS are evaluated at the beginning and end of the study for a global assessment of distress (Multimedia Appendix 2), whereas the MISS-HP is collected for a local assessment of distress (Multimedia Appendix 3).

### Objective Data Collection

**Figure 9:**
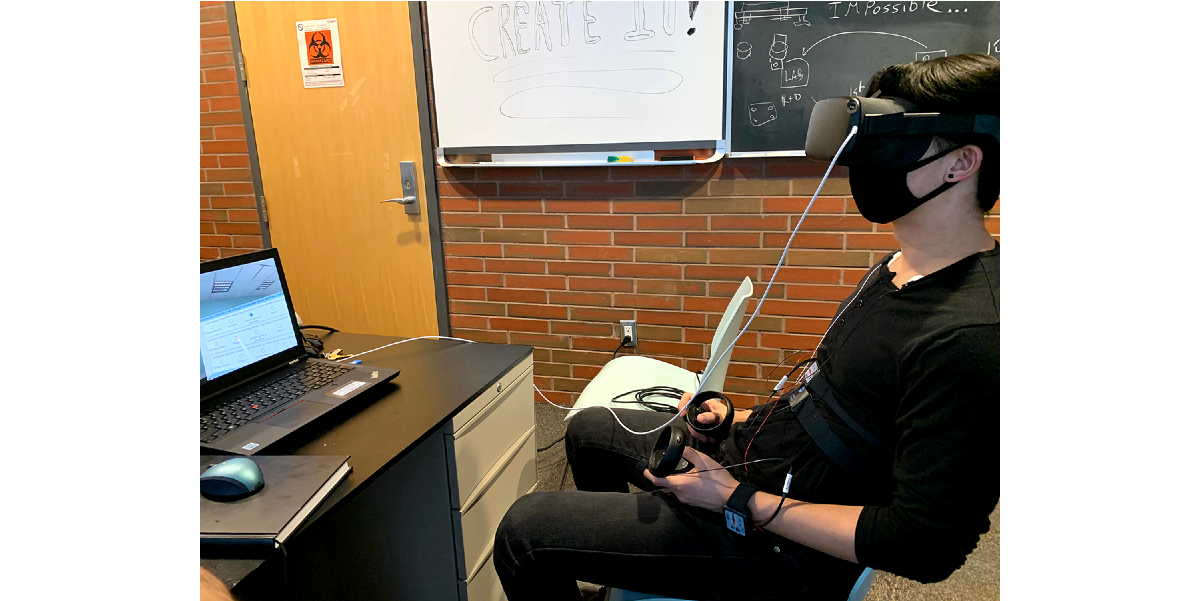
Example of the physical set-up: researcher wearing the head-mounted display, controllers, and Biopac sensors during the virtual reality simulation.

During the virtual reality session, electrocardiogram, electrodermal activity, photoplethysmography, and respiration impedance sensors will be attached to the participant (Figure 9), and these data will be collected with an acquisition and analysis system (MP160, Biopac).

### Virtual Reality Data Analysis

We will create a personalized digital phenotype profile, an objective parameter which will help track individual trends over time which will assist in identifying the efficacy of digital interventions and exposure to immersive virtual reality environments, for each participant using primarily heart rate, respiratory rate, oxygen saturation, and skin conductance (BioNomadix, Biopac) (Table 3). These signal features will be paired with reported psychological measures (scales) for a holistic understanding. Both cardiovascular and cerebrovascular dynamics can be captured with these data by deriving features such as heart rate, respiratory rate, and heart rate variability (which is related to the autonomic nervous system and affect states [19]). The autonomic nervous system is the control system that acts largely unconsciously and regulates bodily functions, including heart rate and respiratory rate. This system is the primary mechanism in control of the fight-or-flight response [19]. We expect that participants in stressful situations will have specific personalized digital phenotype profiles.

**Table 3:** Description of sensors used and physiological signals collected.

Sensor	Description
Galvanic skin response (electrodermal activity)	Galvanic skin response, also known as electrodermal activity, is a sensor that collects the change in the sweat glands. It measures the electrical conduction of the skin. This sensor is commonly reflective of emotional state and arousal such as anxiety
Electrocardiogram	Electrocardiogram is a physiological signal electrocardiogram collects the electrical activity of the heart. We will measure the heart rate using electrocardiogram features that can be extracted can include heart rate variability and R-R intervals
Respiratory impedance	respiration impedance is a physiological signal that collects the respiration of a participant. It can be defined as the mechanical load of the respiratory system to ventilation. We will collect the number of breaths a participant takes
Photoplethysmography	A noninvasive sensor that uses a pulse oximeter to illuminate a light on the skin. By doing so, it measures the blood volume changes respiratory impedance

### Mobile Platform

#### Customized Digital intervention/intelligence Group Platform

We will use the app, which is based on an open-source mobile-based platform, LAMP (Learn, Assess, Manage, Prevent [20,21]), which is fully compliant with patient and participant confidentiality standards and is currently in use at various US, Canadian, and international hospitals [20]. Applicable privacy legislation include the Personal Health Information Protection Act of Ontario and The Personal Information Protection and Electronic Documents Act.

Learn: This module offers health tips and accurate health news from sources the participant can trust [20].

Assess: This module infers the participant’s mood and cognitive status (eg, depression, anxiety, etc) by asking the participant to complete surveys. In addition, this module collects the participant’s phone data (eg, geolocation, phone usage).

Manage: In this module, participants can maintain a journal by writing in a diary or have personalized interventions. However, only the participant can write or read the material in this module since all data collected here is personal and available only to the participant.

Prevent: This module uses a dashboard to post figures and curves summarizing the participant’s mood, cognitive status, and phone data, such as how fast the participant is moving.

The app is compatible with both iOS and Android platforms. It will be available for free download and for use by approved participants (log-in information will be provided after obtaining informed consent), and deidentified data will be stored at Unity Health Toronto. Furthermore, the platform will be tailored to collect information pertinent to the COVID-19 context. The platform collects various forms of active data (while the participant is using the app) and passive data (whether or not the user is actively using the app) [20]. Study participants will have the option of full access to their active and passive data in real time on the phone or web-based dashboard.

### Participant Anonymity

Since participants are required to come to the St. Michael’s Hospital Simulation Centre to complete the virtual reality and wearable components of the study, the study will not be anonymous. Participants will only start using the mobile app after they participate in the virtual reality simulation component of the study. The study coordinator will provide an ID and password by email after participants provide written consent, logistical information (preferred time slot), and demographic information to the study coordinator by email.

### Mobile Data Collection

#### Active Data

The surveys will pop up on fixed days of the week for active data collection (ie, Monday, Wednesday, and Friday), as reminders. The participants are then free to complete the surveys (Table 4). These surveys will allow for monitoring of stress-related symptoms using validated scales—Daily surveys using 15 items taken from the UCLA Loneliness Scale [22] (3 items), PSS [23] (4 items), Generalized Anxiety Disorder [24] scale (2 items), Patient Health Questionnaire [25] (2 items), and MIOS [26] (4 items). Each week, on the weekend, the complete versions of the PSS [23] (10 items), Generalized Anxiety Disorder [24] scale (7 items), Patient Health Questionnaire [25] (9 items), MIOS [26] (10 items). Once during the study, the App Experience Survey (6 items) will be administered.

The content of the Moral Injury Outcome Scale is more specific to reflect the virtual reality scenario, unlike in the mobile study, where the survey is more generalized. The cognitive tests appear similar to electronic games wherein participants have to complete a trail or pattern. We will collect the time and number of errors made by the participants.

#### Passive Data

Passive data (GPS and accelerometer data) are collected automatically while the phone is on, even when the participant is not using the phone (Table 4). The app will access information collected and stored on the phone. Participants can opt out of this feature if they choose.

GPS collects data of the participant’s location data with 3-meter accuracy; however, the participant’s specific location will not be used as data in the study. The app collects GPS data constantly, and data are immediately converted into broad summary metrics, such as the distance per day and or unique locations visited. These data are not monitored in real time, and will not be reviewed by and party.

The accelerometer records triaxial acceleration, which can be used to indicate if the participant is running or walking. These data are not monitored in real time.

Participants may receive an automated alert based on established survey cut-offs at the beginning of the study. After some time, these alerts may be based on the participant’s change in scores compared to their own baseline. The alerts will be simple, for example, “your score today suggests mild anxiety, we suggest exercise, mindfulness practice, etc.” If more than 1 alert is required because more than 1 scale has reached the cut-off, the alert will be given once for all the scales.

**Figure 10:**
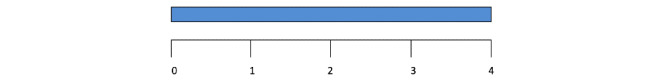
Visual analog scale with a sliding bar for participants to score on the screen of their phones.

For active data, we will use visual analog scales (Figure 10). The scale is linear and continuous for the participant to slide on. The scale is subdivided into varying Likert items according to the questionnaire (eg, a scale with 4 items).

We will use predetermined cut-offs (Table 4) to establish the score for each assessment.

Passive data (eg, GPS, accelerometer) will be collected for the first 2 weeks at the beginning of the study to establish a baseline. Members of the research team will be monitoring the in-person data collection for safety concerns. In addition, participants who are inactive will be contacted to remind them of data collection and patients who are having problems will be contact to give technical support.

**Table 4:** Cut-off values and example notifications.

Assessment and classification				Cut-off values		Example of notification
**Surveys**						
	**General Anxiety Disorder -7**					
		Mild anxiety	5≤score<10		“Based on your scores during the past week, you do not appear clinically anxious/depressed, which is mild/moderate; recommendations include Bibliotherapy, mindfulness, exercise, sleep management”	
		Moderate anxiety	10≤score<15		“Based on your scores during the past week, you do not appear clinically anxious/depressed, which is mild/moderate; recommendations include Bibliotherapy, mindfulness, exercise, sleep management”	
		Severe anxiety	score≥15		“Based on your scores during the past week, you appear clinically anxious/depressed, which is severe; In addition to continued Bibliotherapy, mindfulness, exercise, sleep management”	
	**Perceived Stress Scale**					
		Low	0≤score<14		“Based on your scores during the past week, you do not appear to have increased stress which is mild/moderate; recommendations include Bibliotherapy, mindfulness, exercise, sleep management”	
		Moderate	14≤score<27		“Based on your scores during the past week, you do not appear to have increased stress which is mild/moderate; recommendations include Bibliotherapy, mindfulness, exercise, sleep management”	
		High	score≥27		“Based on your scores during the past 2 week, you appear clinically stressed which is severe; In addition to continued Bibliotherapy, mindfulness, exercise, sleep management”	
	**Patient Health Questionnaire -9**					
		Mild depression	5≤score<10		“Based on your scores during the past week, you do not appear clinically anxious/depressed, which is mild/moderate; recommendations include Bibliotherapy, mindfulness, exercise, sleep management”	
		Moderate depression	10≤score<15		“Based on your scores during the past week, you do not appear clinically anxious/depressed, which is mild/moderate; recommendations include Bibliotherapy, mindfulness, exercise, sleep management”	
		Moderately severe depression	15≤score<20		“Based on your scores during the past week, you appear clinically anxious/depressed, which is severe; In addition to continued Bibliotherapy, mindfulness, exercise, sleep management”	
		Severe depression	score≥20		“Based on your scores during the past week, you appear clinically anxious/depressed, which is severe; In addition to continued Bibliotherapy, mindfulness, exercise, sleep management”	
	Cognitive test: Trails B			>3 minutes or 3 errors		“Your cognition score today is reduced, potential interventions: A) Mindfulness, B) Exercise, C) Sleep habits”
**Passive data**						
	**GPS mobility metrics, call-test logs, latency of response, phone pattern of use, sleep, activity**					
		Moderate change	1 SD to 2 SD from baseline		“Based on your (specify item) scores during the last 2 weeks, you appear to have moderately increased or decreased (specify item); If increased: recommendations include continued exercise; If decreased: recommendations include Bibliotherapy and more exercise”	
		Large change	>2 SD from baseline; direction of change is important		“Based on your (specify item) scores during the last 2 weeks, you appear to have severely increased or decreased (specify item); If increased: recommendations include continued exercise; If decreased: recommendations include Bibliotherapy and more exercise. Also link provided for further help.”	

### Mobile Data Analysis

Smartphones are ubiquitous in our daily lives and incorporate a suite of sensors, such as accelerometer and GPS. In addition, digital platforms can be used to collect active data. This results in a multimodal system on a smartphone device. To objectively analyze the data, preprocessing will include Kalman filtering, GPS data cleaning, and Wavelet transforms, and feature extraction techniques, such as multiview biclustering [27] and statistical and structural features from time-series signals [28], will be used for machine learning (decision tree, logistic regression, and support vector machine) to find associations between passive and active data. These models are simple, consume low power, and have the capability of explainable artificial intelligence to understand our machine learning models better [29]. We plan to extend the machine learning models to long short-term memory and convolutional neural networks due to the popularity and effectiveness of the models in mobile data analysis [28].

### Analysis Approach

**Figure 11:**
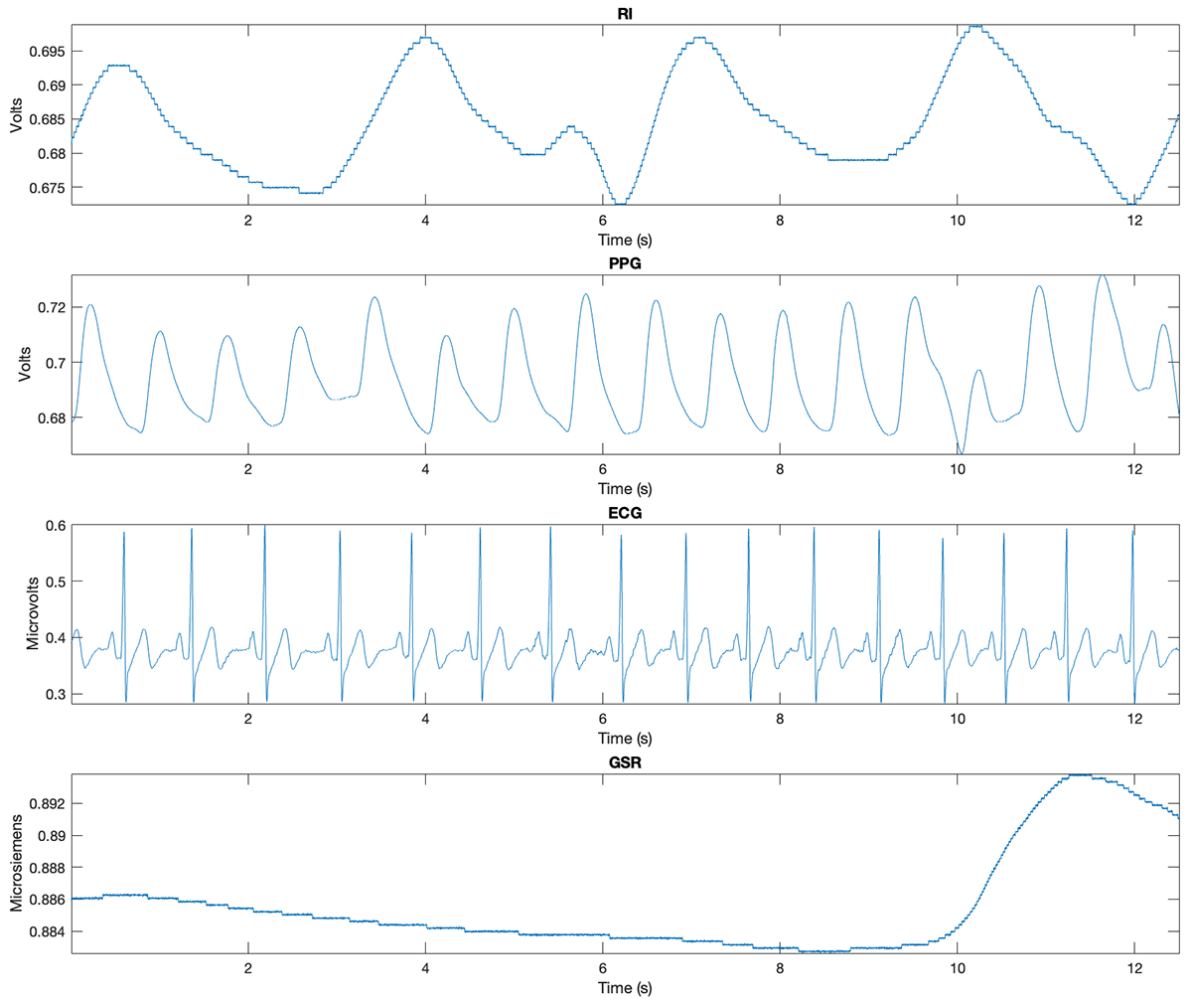
Physiological signal examples: respiration impedance (RI), photoplethysmography (PPG), electrocardiogram (ECG), and galvanic skin response (GSR).

Signals (Figure 11; Multimedia Appendix 4) will be collected with a sampling frequency of 2000 Hz. The software used to collect the data from Biopac performs all internal calculations to the accuracy defined by the IEEE format for double-precision floating-point numbers and stores the results of those calculations in double-precision floating-point format; this format assigns 8 bytes to all numbers in calculations or resulting from calculations [30]. Biopac equipment has an interface to interact with the virtual reality scenario; thus, we have markers for the sessions, such as the start and end of the session and when the user answers a question.

Numeric programming computing software will be used to conduct the analysis. The signals will be collected in a raw format. Preprocessing, feature extraction, and machine learning will be applied to the data. Preprocessing involves techniques such as low-pass filters, high-pass filters, and notch filters to remove noise. The filtered signal will then be passed along to extract features. Feature extraction will involve extracting attributes of the signals to be better represented. Techniques for feature extraction include handcrafted features, such as statistical values. Lastly, machine learning techniques such as decision tree, random forest, and logistic regression will be used for classification. These techniques are chosen due to their ability to manage small sample sizes. Once familiar with the newly acquired data set, we will reflect on a new approach for analysis.

## Results

As of March 20, 2021, the research ethics board at St. Michael’s Hospital has approved the study (UHTDTS25377). This project was funded on November 20, 2020.

## Discussion

As previously explored, this research will develop and validate the use of digital platforms, specifically virtual reality and mobile apps to understand the continuum of moral distress that leads to moral. Demonstration of feasibility will lead to future studies to examine the efficacy of these digital interventions.

We hypothesize that features of the physiological data will help to identify mental health disorders and distress within users. For example, many studies [31,32] have looked at the impact of stress and burnout in health care workers due to the COVID-19 pandemic.

Modern signal processing and classification tools will be used to find associations between the data and mental health surveys. We expect to discover identifiers within physiological signals that reflect distress and other psychological disorders in the short- and long-term fluctuations of the passive data response related to mental health.

Given the pilot project’s aims, the number of participants (10-15 health care providers) was chosen with a view toward demonstrating feasibility while taking into consideration the availability of participants during the COVID-19 pandemic.

Determining the feasibility and utility of virtual reality environments to aid health care workers in reducing distress and becoming resilient in morally challenging and complex environments, by using physiological data in a personalized digital phenotype profile with mobile platforms will lead to future work on the implementation of a larger scale study.

## Appendix

Multimedia Appendix 1 Requirements for LAMP (Learn, Assess, Manage, Prevent) mobile app servers.

Multimedia Appendix 2 Moral Injury Outcome Scale and Perceived Stress Scale.

Multimedia Appendix 3 Adapted Moral Injury Symptom Scale: Healthcare Professionals version.

Multimedia Appendix 4 Additional physiological signals.

## Abbreviations

IPQ: Igroup presence questionnaire
MIOS: Moral Injury Outcome Scale
MISS-HP: Moral Injury Symptom Scale: Healthcare Professional version
PSS: Perceived Stress Scale
