# Supplementary material for: Digital Interventions to Reduce Distress Among Health Care Providers at the Frontline: Protocol for a Feasibility Trial
Source: JMIR Res Protoc. 2022 Feb 16;11(2):e32240. doi: 10.2196/32240 (PMC8852627; doi:10.2196/32240)
Supplement: Multimedia Appendix 1 [file resprot_v11i2e32240_app1.pdf]

# Multimedia Appendix 1: Requirements for LAMP (Learn, Assess, Manage, Prevent) mobile application servers

| Component | Requirements |
|---|---|
| Virtual machine server | t3.medium (Ubuntu 18+, 2 CPU, 4 Gb RAM[a], 30 GB solid state drive) |
| Database server | t3.medium (Ubuntu 18+, 2 CPU, 4 Gb RAM[a], 20 GB solid state drive) |

[a]RAM size may increase while moving to production. Therefore, load tests need to be done after a particular release.
