# Supplementary material for: Digital Interventions to Reduce Distress Among Health Care Providers at the Frontline: Protocol for a Feasibility Trial
Source: JMIR Res Protoc. 2022 Feb 16;11(2):e32240. doi: 10.2196/32240 (PMC8852627; doi:10.2196/32240)
Supplement: Multimedia Appendix 2 [file resprot_v11i2e32240_app2.pdf]

Multimedia Appendix 2: Moral Injury Outcome Scale and Perceived Stress Scale

Perceived Stress Scale

The Perceived Stress Scale (PSS) is a classic stress assessment instrument. The questions in this scale ask about your feelings and thoughts. In each case, you will be asked to indicate how often you felt or thought a certain way.

Please answer the questions with a focus of your feelings and thoughts during the last month

For each question choose from the following alternatives:
0 - never 1 - almost never 2 - sometimes 3 - fairly often 4 - very often

________ 1. In the last month, how often have you been upset because of something that happened unexpectedly?

________ 2. In the last month, how often have you felt that you were unable to control the important things in your life?

________ 3. In the last month, how often have you felt nervous and stressed?

________ 4. In the last month, how often have you felt confident about your ability to handle your personal problems?

________ 5. In the last month, how often have you felt that things were going your way?

________ 6. In the last month, how often have you found that you could not cope with all the things that you had to do?

________ 7. In the last month, how often have you been able to control irritations in your life?

________ 8. In the last month, how often have you felt that you were on top of things?

________ 9. In the last month, how often have you been angered because of things that happened that were outside of your control?

________ 10. In the last month, how often have you felt difficulties were piling up so high that you could not overcome them?

Brief Moral Injury Outcome Scale*

<u>Instructions</u>: This questionnaire asks about experiences you may have had after a very stressful experience in which:

- **You did something (or failed to do something) that went against your moral code or values (e.g., you harmed someone or failed to protect someone from harm), or**
- **You saw someone (or people) do something or fail to do something that went against your moral code or values (e.g., you witnessed cruel behavior), or**
- **You were directly affected by someone doing something or failing to do something that went against your moral code or values (e.g., being betrayed by someone you trusted).**

Have you had an experience (or experiences) as described above? YES NO

If yes, please answer questions A-C while thinking about <u>the worst event that currently bothers you the most</u>. This could be one of the examples above, or some other very stressful experience that went against your core values.

A. Did the event involve something you did or failed to do?

_____Yes _____No

B. Did the event involve observing someone else acting (or failing to act)?

_____Yes _____No

C. Did the event involve being directly impacted by someone else (or people) acting (or failing to act)?

_____Yes _____No

Keeping this worst event in mind, please read each of these statements and circle one of the numbers to the right to indicate how much you would agree with the statement <u>in the past month</u>.

In the past month, how strongly would you <u>agree with</u> the following statements:	*Strongly Disagree*	*Disagree*	*Neither Agree or Disagree*	*Agree*	*Strongly Agree*
1. I blame myself.	0	1	2	3	4
2. I have lost faith in humanity.	0	1	2	3	4
3. People would hate me if they really knew me.	0	1	2	3	4
4. I have trouble seeing goodness in others.	0	1	2	3	4
5. I am disgusted by what happened.	0	1	2	3	4
6. I keep myself from having success.	0	1	2	3	4
7. There is no higher power.	0	1	2	3	4
8. I lost trust in others.	0	1	2	3	4
9. I am quick to be angry.	0	1	2	3	4
10. I am not the good person I thought I was.	0	1	2	3	4

Please circle a number below that represents how much these experiences have made it hard for you to take care of yourself (e.g., do pleasurable things, exercise, eat properly), or to be effective in your job, in school, or seeking employment, or to get along with other people?

Not at All			Somewhat			Extremely
0	1	2	3	4	5	6

**The Brief Moral Injury Outcome Scale* (2020). Litz, B.T., Phelps, A., Frankfurt, S., Murphy, D. Nazarov, A. Houle, S., Levi-Belz, Y., Zerach, G., Dell, L., Hosseiny, F., and the members of the *Moral Injury Outcome Scale (MIOS) Consortium.* MIOS consortium activities were supported in part by contributions from Veterans Affairs Australia, Phoenix Centre for Posttraumatic Mental Health, and the Canadian Centre of Excellence on PTSD and Related Mental Health Conditions.
