# Supplementary material for: Digital Interventions to Reduce Distress Among Health Care Providers at the Frontline: Protocol for a Feasibility Trial
Source: JMIR Res Protoc. 2022 Feb 16;11(2):e32240. doi: 10.2196/32240 (PMC8852627; doi:10.2196/32240)
Supplement: Multimedia Appendix 3 [file resprot_v11i2e32240_app3.pdf]

Multimedia Appendix 3: Adapted Moral Injury Symptom Scale - Healthcare Professionals Version (MISS-HP)

In the current situation, how strongly would you agree with the following statement:	*Strongly Disagree*	*Disagree*	*Neither Agree or Disagree*	*Agree*	*Strongly Agree*
**I feel betrayed by other health professionals whom I once trusted.**	**0**	**1**	**2**	**3**	**4**
**I have a good sense of what makes my life meaningful as a health professional.**	**0**	**1**	**2**	**3**	**4**
**Most people with whom I work as a health professional are trustworthy.**	**0**	**1**	**2**	**3**	**4**

In the current situation, how strongly would you agree with the following statement:	*Strongly Disagree*	*Disagree*	*Neither Agree or Disagree*	*Agree*	*Strongly Agree*
**I am troubled by having acted in ways that violated my own morals or values.**	**0**	**1**	**2**	**3**	**4**
**I feel ashamed about what I've done or not done when providing care to my patients.**	**0**	**1**	**2**	**3**	**4**

In the current situation, how strongly would you agree with the following statement:	*Strongly Disagree*	*Disagree*	*Neither Agree or Disagree*	*Agree*	*Strongly Agree*
**I feel guilt over failing to save someone from being seriously injured or dying.**	**0**	**1**	**2**	**3**	**4**
**All in all, I am inclined to feel that I'm a failure in my work as a health professional.**	**0**	**1**	**2**	**3**	**4**

In the current situation, how strongly would you agree with the following statement:	*Strongly Disagree*	*Disagree*	*Neither Agree or Disagree*	*Agree*	*Strongly Agree*
**I have forgiven myself for what's happened to me or to others whom I have cared for.**	**0**	**1**	**2**	**3**	**4**
**Compared to before I went through these experiences, my religious/spiritual faith has strengthened.**	**0**	**1**	**2**	**3**	**4**
**I sometimes feel God is punishing me for what I've done or not done while caring for patients.**	**0**	**1**	**2**	**3**	**4**
