# Supplementary material for: Digital Interventions to Reduce Distress Among Health Care Providers at the Frontline: Protocol for a Feasibility Trial
Source: JMIR Res Protoc. 2022 Feb 16;11(2):e32240. doi: 10.2196/32240 (PMC8852627; doi:10.2196/32240)

Multimedia Appendix 4: Additional physiological signals

Figure 1. ECG with event markers displayed.

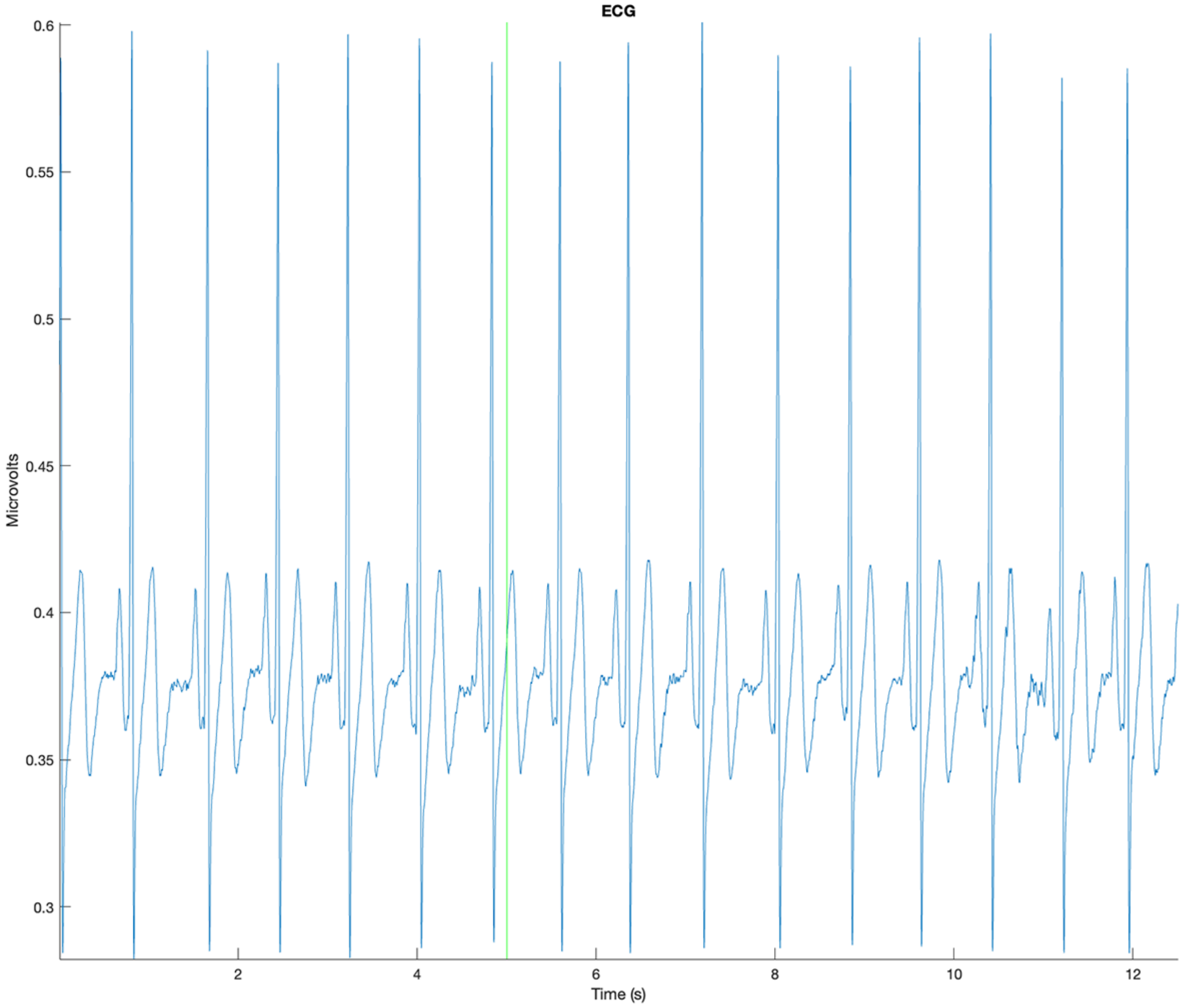

Figure 2. RI with event markers displayed.

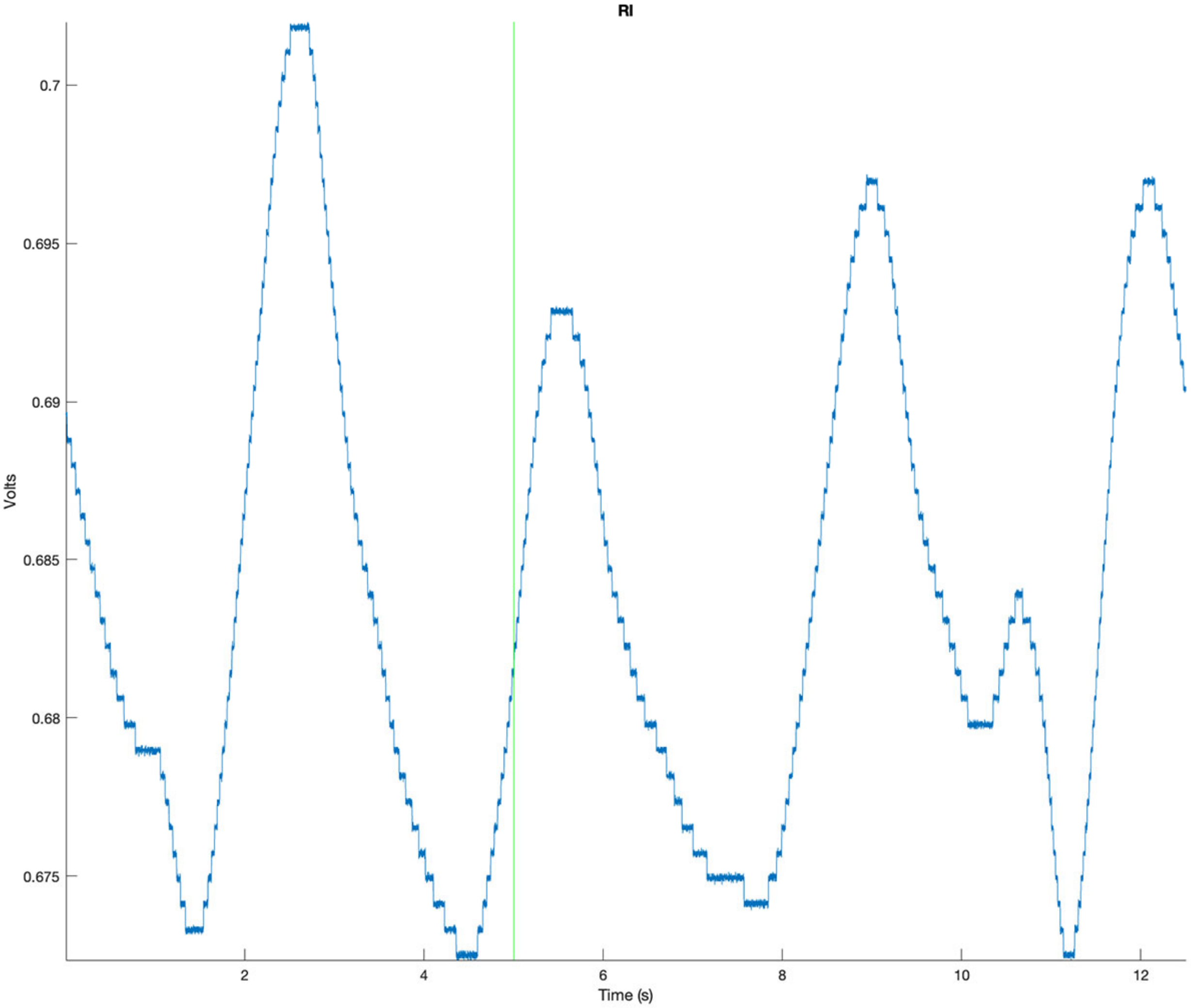

Supplement: Multimedia Appendix 4 [file resprot_v11i2e32240_app4.pdf]
